# A computational study on the effects of fast-rising voltage on ionization fronts initiated in sub-mm air and CO_2_ gaps

**DOI:** 10.1038/s41598-024-51727-y

**Published:** 2024-01-12

**Authors:** Timothy Wong, Igor Timoshkin, Scott MacGregor, Mark Wilson, Martin Given

**Affiliations:** High Voltage Technologies (HVT) Research Group, Department of Electronic and Electrical Engineering, University of Strathclyde, Glasgow, G1 1XQ Scotland, UK

**Keywords:** Electrical and electronic engineering, Plasma physics

## Abstract

Gas discharge and breakdown phenomena have become increasingly important for the development of an ever-growing number of applications. The need for compact and miniaturized systems within power, pulsed power, semiconductor, and power electronic industries has led to the imposing of significant operating electric field stresses on components, even within applications with low operating voltages. Consequently, the interest in gas discharge processes in sub-millimeter and microscale gaps has grown, as the understanding of their initiation and propagation is critical to the further optimization of these technologies. In this work, a computational study of primary ionization fronts has been conducted, which systematically investigated the role of voltage rate-of-rise in point-plane and point-point electrode geometries with an inter-electrode gap maintained at 250 $\upmu$m and a needle radius of 80 $\upmu$m. Using the hydrodynamic approach with the local mean energy approximation, along with simplified plasma chemistry, simulations have been performed under positive and negative ramp voltages, rising at 50, 25, 16.67, 12.5, and 10 kV/ns in synthetic air and in pure CO_2_. Results on the developed electric field, electron densities, and propagation velocities are presented and discussed. Effects on the cathode sheath thickness scaling with voltage rate-of-rise have been additionally analyzed, the mechanisms behind these effects and their potential impacts are discussed. The work conducted in this study contributes towards an increased understanding of the gas discharge process, under fast-transients and nonuniform electric fields, with relevance to microelectromechanical, power, and pulsed power system design.

## Introduction

In recent times, there has been increasing interest in gas discharge processes in various gases, brought on by the requirements for miniaturization in several areas, for instance, in the power, pulsed power, semiconductor, and power electronic industries. While issues surrounding gas breakdown were once mainly confined to large-scale high voltage (HV) electrical systems, the drive towards miniaturization and compact technology and has led to two phenomena. Firstly, a growing number of low-voltage devices find themselves squarely within the domain of electrical breakdown due to new restrictions on physical dimensions, where in the past, the issue could be largely ignored. Secondly, HV systems which had previously been designed for particular ranges of electric field, may now face new and elevated levels of electrical stress, posing new challenges for insulation design and coordination. Some examples of applications include the prevention of breakdown in microelectromechanical systems (MEMS) or novel power electronic devices^1^, and equipment used in pulsed power or low temperature plasma (LTP) systems, e.g., plasma closing switches^2^, electrostatic precipitation systems^3^, and for pulsed electric field (PEF) treatment^4^.

On this account, the continued development and optimization of these technologies is heavily reliant upon a deeper understanding of elementary gas discharge processes. In practice, processes under nonuniform and time-varying electric fields are of particular importance, for which the understanding is currently limited. Available literature on sub-mm gaps (but larger than that of the lower Paschen limit of $\lesssim$ 15 $\upmu$m at 1 atm^5^) is scarce, particularly for sub-mm gaps involving nonuniform electric fields combined with fast-rising voltages. Simulation studies that investigated the role of the voltage rise time have been conducted in the past^6–8^, but these were under long, oil-filled, millimeter gaps over full impulses as opposed to short gas-filled sub-mm gaps with specific focus on the rising edge. Nevertheless, the authors showed that the voltage rate-of-rise may affect characteristics such as the streamer radius and velocity when in oil. Experimental studies which were conducted under a similar configuration to the present work include those by Hogg et al.^9,10^, where the authors investigated the breakdown of bottled air in sub-mm point-plane gaps down to 250 $\upmu$m, pressurized between 0.1 and 0.35 MPa. They reported an increasing breakdown strength with the decrease in gap distance for positive energization, such that the positive breakdown voltage exceeded that of its negative counterpart for smaller gaps. This contrasted with longer gaps (> 4 mm), where the opposite was generally observed: positive energization typically induced breakdown at a lower voltage.

In work by Liu et al.^11^, field-time breakdown characteristics were investigated under fast-rising nanosecond impulses, in a 100 $\upmu$m needle-plane gap and with a needle tip radius of approximately 80 $\upmu$m. Separate gaps filled with air, CO_2_, and N_2_ were tested, from which the authors consistently observed higher breakdown voltages at shorter breakdown times in CO_2_ compared to the two other gases. This departed from classical streamer inception theory based on solely on Townsend coefficients, which in contrast, had been shown to fit experimental data under longer gaps well, e.g., as shown by Kumar et al.^12^. There was also a marked difference between the field-time characteristics obtained in Liu et al.^11^ and the analytical streamer transition (Meek) criterion, to which the authors attribute mainly to statistical time lag. Moreover, both Hogg et al.^10^ and Kumar et al.^12^ observed higher stability in the negative breakdown of CO_2_ compared to positive breakdown, which had been attributed to negative space charge and corona stabilization effects^10^, or to the existence of initial electrons^12^. The effects observed in these studies emphasized the degree to which processes within this regime remain unknown, and that a deeper understanding must be gained to further develop systems that are reliant upon short-gap gas discharge processes.

In this study, the initial discharge phase consisting of the propagation of a primary ionization front has therefore been computationally investigated. Using point-plane and point-point electrode gaps of 80 $\upmu$m radius and 250 $\upmu$m separation, the characteristics of the primary ionization front in synthetic air and in CO_2_ have been studied, under fast-rising ramp voltages of different rates-of-rise, and for both positive and negative energization. The linearly rising voltages can be considered as an approximation for *overstressed* or *overvolted* breakdown on the rising slope of an impulse, as commonly featured in pulsed power systems. These terms refer to breakdown occurring at electric fields or voltages higher than that of the static breakdown values, and is characteristic of pulsed breakdown. The *overvoltage* is defined as the difference between the impulsive breakdown voltage and the static breakdown voltage^13^. The choice to study CO_2_ was further informed by its importance in some chemical processing applications, such as CO_2_ splitting^14^, and for its potential to be used in gas mixtures that may act as a replacement for the potent greenhouse gas, SF_6_, within gas-insulated power and pulsed power equipment^15–17^.

## Gas discharge model

This section details the mathematical model employed in this work to investigate the gas discharge process. Namely, the *hydrodynamic* or *fluid* approach has been used, which seeks self-consistent solutions to the spatiotemporal evolution of a set charged particle species by approximating them as continuous charge densities. These densities undergo advection, diffusion, and reaction, under the influence of both an external field and their own space charge induced electric fields. The hydrodynamic approach has gained popularity in recent times, favored for its faster computation times as compared with more fundamental descriptions, such as particle-based kinetic methods. There are, however, known limitations to the fluid approach, see for instance the regions of validity as outlined by Kolobov and Arslanbekov^18^. It is remarked that based solely on the characteristic length, *L*, of the domain and gas pressure used here, the present configuration remains within the region of validity for the hydrodynamic approach. That is, the mean free path of electrons in both atmospheric air and CO_2_ under the simulated conditions is far shorter than *L*. It is, however, also important to note that the wavefront evolutions modelled in this work take place over picosecond timescales, placing the characteristic times of the discharges close to the relaxation time of the electron energy distribution function (EEDF). Based on analysis by Zhu et al.^19^, the fast-discharge conditions considered in the present analyses are close the limit of validity for the hydrodynamic approach, where a kinetic approach may begin to become necessary. However, considering the uncertainty in the exact position and nature of this validity criterion (i.e, the boundary between these two approaches); the hydrodynamic approach is considered to be valid for the conditions modelled in the present paper. As further support for the validity of the fluid approach, the reader is referred to a combined experimental/simulation study by Höft et al.^20^, where reasonable agreement between fluid-simulated and experimentally imaged primary ionization waves was found. Those studied were developed under similarly fast-rising voltages as the present work, and occurred over comparably short (picosecond) timescales. Comparison of the resultant wavefront velocities arising from this work to those experimentally measured by Tardiveau et al.^21^ , also under voltages rising on the order of kV/ns, supports the suggestion that the fluid model may remain a reasonable approximation of reality near this limit. The exploration of kinetic effects is left as a subject for future work to build upon the results presented here.

### Drift-diffusion equations

Using the hydrodynamic approximation for gas discharges, and for a chemical species $i \in N$, where *N* is the set of all tracked species, the charge density evolves in space and time with:

1 $$\begin{aligned} \partial _t n_i + \nabla \cdot \mathbf {\Gamma }_i = S_i, \end{aligned}$$

where $\partial _t$ represents the time derivative, $n_i$ is the volumetric density, and the total flux $\mathbf {\Gamma }_i$ is characterized by the (positive) mobility, $\mu _i$, and the diffusion coefficient, $D_i$, following:

2 $$\begin{aligned} \mathbf {\Gamma}_i = -\textrm{sgn}(q_i) n_i \mu_i \nabla \varphi - D_i \nabla n_i, \end{aligned}$$

given that $q_i$ is the signed charge of species *i*, and $\varphi$ is the scalar potential field. The charge densities are coupled to the electric field through the Poisson equation:

3 $$\begin{aligned} -\nabla \cdot \left( \varepsilon \nabla \varphi \right) =\sum _{j \in N} q_j n_j, \end{aligned}$$

where $\varepsilon$ is the permittivity of the medium. In the case of gas, this is assumed to be equal to the vacuum permittivity. A self-consistent solution can be found by solving (1) and (3) simultaneously with appropriate boundary and initial conditions, those used in this study are described later in the section “Domain and boundary conditions”.

### Plasma chemistry

This study considered two gases: synthetic air (80/20% N_2_/O_2_) and pure CO_2_. For air, the simplified plasma chemistry set following Pancheshnyi and Starikovskii^22^ has been used, while those provided by Aerts et al.^14^ were employed for CO_2_. Electronic reaction rates and transport coefficients were computed using BOLSIG+^23^, from Phelps’ collision cross sections^24^, while all heavy species were considered non-diffusive and immobile over the simulated timescales. The reactions and rate coefficients are tabulated in Tables 1 and 2 for air and CO_2_, respectively.

**Table 1:** Table of included chemical reactions for air.

Reaction number	Description	Reaction	Rate	Unit	References
$R_1$	Impact ionization (15.6 eV)	$\text {e} + N_2 \rightarrow N_2^+ + \text {e} + \text {e}$	BOLSIG+	m^3^s^-1^	^23^
$R_2$	Impact ionization (18.8 eV)	$\text {e} + N_2 \rightarrow N_2^+ + \text {e} + \text {e}$	BOLSIG+	m^3^s^-1^	^23^
$R_3$	Impact ionization	$\text {e} + \text {O}_2 \rightarrow \text {O}_2^+ + \text {e} + \text {e}$	BOLSIG+	m^3^s^-1^	^23^
$R_4$	Attachment	$\text {e}+\text {O}_2+\text {O}_2 \rightarrow \text {O}_2^- + \text {O}_2$	$f({\bar{\varepsilon }})$	m^6^s^-1^	^23^
$R_5$	Rapid $\text {O}_2^+$ production	$N_2^+ + N_2 + \text {M} \rightarrow N_4^+ + \text {M}$	$5\times 10^{-41}$	m^6^s^-1^	^22^
$R_6$	Rapid $\text {O}_2^+$ production	$N_4^+ + \text {O}_2 \rightarrow \text {O}_2^+ + N_2 + N_2$	$2.5\times 10^{-16}$	m^3^s^-1^	^22^
$R_7$	Rapid $\text {O}_2^+$ production	$N_2^+ + \text {O}_2 \rightarrow \text {O}_2^+ + N_2$	$6\times 10^{-17}$	m^3^s^-1^	^22^
$R_8$	$\text {O}_2^+$ to $O_4^+$ conversion	$\text {O}_2^+ + N_2 + N_2 \rightarrow \text {O}_2^+N_2 + N_2$	$9\times 10^{-43}$	m^6^s^-1^	^22^
$R_9$	$\text {O}_2^+$ to $O_4^+$ conversion	$\text {O}_2^+N_2+N_2 \rightarrow \text {O}_2^++N_2+N_2$	$4.3\times 10^{-16}$	m^3^s^-1^	^22^
$R_{10}$	$\text {O}_2^+$ to $O_4^+$ conversion	$\text {O}_2^+N_2+\text {O}_2 \rightarrow O_4^+N_2$	$1\times 10^{-15}$	m^3^s^-1^	^22^
$R_{11}$	$\text {O}_2^+$ to $O_4^+$ conversion	$\text {O}_2^++\text {O}_2+\text {M} \rightarrow O_4^++\text {M}$	$2.4\times 10^{-42}$	m^6^s^-1^	^22^
$R_{12}$	Electron-ion recombination	$\text {e}+O_4^+ \rightarrow \text {O}_2+\text {O}_2$	$f({\bar{\varepsilon }})$	m^3^s^-1^	^22^
$R_{13}$	Electron-ion recombination	$\text {e}+\text {O}_2^+ \rightarrow \text {O}+ \text {O}$	$f({\bar{\varepsilon }})$	m^3^s^-1^	^22^
$R_{14}$	Ion-ion recombination	$\text {O}_2^- + O_4^+ \rightarrow \text {O}_2+\text {O}_2+\text {O}_2$	$1\times 10^{-13}$	m^3^s^-1^	^22^
$R_{15}$	Ion-ion recombination	$\text {O}_2^-+O_4^++\text {M} \rightarrow \text {O}_2+\text {O}_2+\text {O}_2+\text {M}$	$2\times 10^{-37}$	m^6^s^-1^	^22^
$R_{16}$	Ion-ion recombination	$\text {O}_2^- + \text {O}_2^+ + \text {M} \rightarrow \text {O}_2 + \text {O}_2 + \text {M}$	$2\times 10^{-37}$	m^6^s^-1^	^22^
$R_{17}$	Excitation/emission	$\text {e}+N_2 \rightarrow \text {e}+N_2+\gamma$	Zheleznyak.	−	^25^
$R_{18}$	Photoionization	$\gamma + \text {O}_2 \rightarrow \text {e}+\text {O}_2^+$	Zheleznyak.	−	^25^

M denotes $\text {O}_2$ and $\text {N}_2$. BOLSIG+ represents tabulated data computed using bolsig^23^. $f({\bar{\varepsilon }})$ indicates that the reaction rate is an empirical function of the local mean energy.

**Table 2:** Table of included chemical reactions for $\text {CO}_2$.

Reaction number	Description	Reaction	Rate	Unit	References
$R_1$	Impact ionization	$\text {e} + \text {CO}_2 \rightarrow \text {CO}_2^{+}+\text {e}+\text {e}$	BOLSIG+	m^3^s^-1^	^23^
$R_2$	Dissociation	$\text {e}+\text {CO}_2 \rightarrow \text {CO} +\text {O}+ \text {e}$	$5\times 10^{-17}$	m^3^s^-1^	^14^
$R_3$	Attachment	$\text {e}+\text {CO}_2 \rightarrow \text {CO}+\text {O}^-$	BOLSIG+	m^3^s^-1^	^23^
$R_4$	Dissociation	$\text {e}+\text {O}_3 \rightarrow \text {O} + \text {O}_2 + \text {e}$	$2\times 10^{-15}$	m^3^s^-1^	^14^
$R_5$	Dissociation	$\text {e}+\text {O}_2 \rightarrow \text {O}+\text {O}+\text {e}$	$2\times 10^{-15}$	m^3^s^-1^	^14^
$R_6$	Dissociation	$\text {e}+\text {O}_2 \rightarrow \text {O}+\text {O}^-$	$4\times 10^{-17}$	m^3^s^-1^	^14^
$R_7$	Attachment	$\text {e}+\text {O}_2+\text {M} \rightarrow \text {O}_2^- +\text {M}$	$3\times 10^{-42}$	m^6^s^-1^	^14^
$R_8$	Ion-neutral reaction	$\text {O}^-+\text {CO} \rightarrow \text {CO}_2+\text {e}$	$5.5\times 10^{-16}$	m^3^s^-1^	^14^
$R_9$	Ion-neutral reaction	$\text {O}^-+\text {O}_2 \rightarrow \text {O}_3 +\text {e}$	$1\times 10^{-18}$	m^3^s^-1^	^14^
$R_{10}$	Ion-neutral reaction	$\text {O}^- + \text {O}_3 \rightarrow \text {O}_2 + \text {O}_2 + \text {e}$	$3\times 10^{-16}$	m^3^s^-1^	^14^
$R_{11}$	Electron-ion recombination	$\text {e}+\text {CO}_2^+ \rightarrow \text {CO} + \text {O}$	$6.5\times 10^{-13}$	m^3^s^-1^	^14^
$R_{12}$	Ion-ion recombination	$\text {O}_2^- + \text {CO}_2^+ \rightarrow \text {CO} + \text {O}_2 + \text {O}$	$6\times 10^{-13}$	m^3^s^-1^	^14^
$R_{13}$	Neutral reaction	$\text {O}+\text {O}+\text {M} \rightarrow \text {O}_2 +\text {M}$	$1.04\times 10^{-45}$	m^6^s^-1^	^14^
$R_{14}$	Neutral reaction	$\text {O}+\text {O}_2+\text {M} \rightarrow \text {O}_3+\text {M}$	$4.42\times 10^{-46}$	m^6^s^-1^	^14^
$R_{15}$	Neutral reaction	$\text {O}+\text {O}_3 \rightarrow \text {O}_2 + \text {O}_2$	$7.56\times 10^{-18}$	m^3^s^-1^	^14^
$R_{16}$	Neutral reaction	$\text {O}+\text {CO}+\text {M} \rightarrow \text {CO}_2 + \text {M}$	$1.11\times 10^{-47}$	m^6^s^-1^	^14^
$R_{17}$	Neutral reaction	$\text {O}_3+\text {M} \rightarrow \text {O}_2 + \text {O} + \text {M}$	$1.16\times 10^{-32}$	m^6^s^-1^	^14^

BOLSIG+ represents tabulated data computed using bolsig^23^. $f({\bar{\varepsilon }})$ indicates that the reaction rate is an empirical function of the local mean energy.

$R_{17}$ and $R_{18}$ of Table 1 correspond to photoionization, which is described in more detail within the section named photoionization and pre-ionization. The source terms of (1) were then computed following:

4 $$\begin{aligned} S_i = \sum _{j=1}^r \left( h_j k_j \prod _{m \in R} n_m \right) , \end{aligned}$$

where $k_j$ is the reaction rate for reaction $j \in r$, *r* being the set of all reactions. $h_j$ is +1 or -1 depending if the reaction is a source or sink, while *R* is the set of all reactants partaking in reaction *j*. $n_m$ is therefore the density of the *m*-th reactant.

### Photoionization and pre-ionization

It is known that an external source of electrons must be present ahead of positive ionization fronts for their successful development and sustained propagation. In air, this source is widely considered to be photoionization^26^ due to the excitation and subsequent radiative de-excitation of N_2_ molecules which ionize O_2_ (following R_17_ and R_18_ of Table 1). The present model includes this process using Zheleznyak’s model^25^, approximated using the three-term Helmholtz approach described by Bourdon et al.^27^. The photoelectron source term is therefore given by:

5 $$\begin{aligned} \nabla ^2 S_{ph,j} - \left( p_{\text {O}_2}\lambda _j\right) ^2 S_{ph,j}=-\left( A_j p_{\text {O}_2}^2\frac{p_q}{p+p_q}\xi \frac{\nu _u}{\nu _i}\right) S_{ion}, \nonumber \\ S_{ph} = \sum _j S_{ph,j}, \end{aligned}$$

for *j* = 1, 2, 3, and is included as a source in Eq. (1). Here, *p* is the gas pressure, $p_{\text {O}_2}$ is the partial pressure of oxygen, and $p_q$ is the collisional quenching pressure of nitrogen, which accounts for non-radiative de-excitation processes. The fitting parameters $A_j$, $\lambda _j$ and $\xi \nu _u/\nu _i$ used throughout this study follows those given by Bagheri et al.^28^. In air, it is also assumed that a pre-ionization level of $10^9$ m^-3^ (electrons and $\text {N}_2^+$ ions) exists in the domain, which represents a typical value of the background ionization level^29^.

In CO_2_, the role of photoionization remains largely unknown. Bagheri et al.^30^ suggested that photoionization in CO_2_ would be negligible, based on previous experimental measurements. To date, there has been no significant findings to suggest otherwise. To alleviate the computational challenge of simulating discharges with a low electron source, an elevated level of pre-ionization ($10^{13}$ m^-3^, electrons and $\text {CO}^{+}_2$) has therefore been incorporated for CO_2_ simulations^30^. According to previously conducted computational tests^30^, streamer discharges in CO_2_ do not exhibit significant sensitivity to the level of background pre-ionization, through it may be important to branching behavior. However, the present study investigated a short gap of only 250 $\upmu$m, a distance for which branching is unlikely to be relevant. It was therefore concluded that this approximation would not substantially affect the obtained characteristics of the discharge evolution.

### Local mean energy approximation

While the local field approximation (where the transport parameters are a function only of the local electric field strength) can adequately describe non-thermal gas discharges in some scenarios, this approximation becomes less applicable in high or nonuniform field regions^31^, such as near solid boundaries, sharp electrodes, or short gaps. To expand the range of validity of the present model, the local mean energy approximation has instead been used in this work. This incorporates an additional balance equation for the electron energy, which explicitly accounts for energy losses within chemical reactions, and the energy change relating to the field heating and cooling of electrons, given by:

6 $$\begin{aligned} \partial _t n_\varepsilon + \nabla \cdot \mathbf {\Gamma }_\varepsilon = {\bar{e}}\mathbf {\Gamma }_e \cdot \nabla \varphi -\sum _{j=1}^n \left( \Delta E_j k_j \prod _{m \in R} n_m \right) , \end{aligned}$$

where $n_\varepsilon$ is the electron energy density, from which the local electron energy, $\bar{\varepsilon }$ is found from $\bar{\varepsilon }= n_\varepsilon /n_e$. As before, $n_m$ is the density of reactant *m* in the set *R*. The symbol ${\bar{e}}$ is the elementary charge, while $\Delta E_j$ is the electron energy change during reaction *j*. Transport coefficients were then set to be a function of $\bar{\varepsilon }$ rather than of the local electric field magnitude.

### Domain and boundary conditions

**Figure 1:**
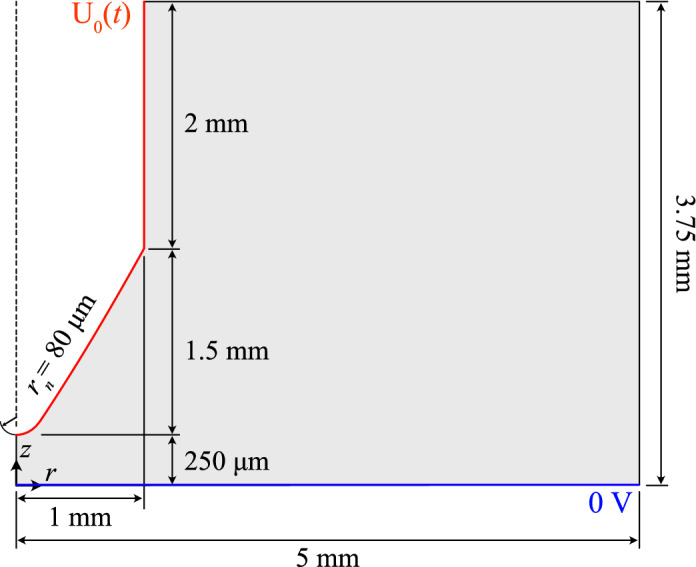
Diagram of the computational domain for needle-plane simulations. For needle-needle, the plane electrode was replaced with a second, identical and mirrored, needle electrode with tips placed at $z = \pm$ 125 $\upmu$m.

The domain used for point-plane simulations is shown in Fig. 1. The needle was formed of a hyperbolic segment that with rotational symmetry about *r* = 0, and had a tip radius of 80 $\upmu$m. To better approximate a practical needle geometry, the hyperbola was connected to a straight segment representing the outer cylindrical face of the needle, which had a radius of 1 mm. The bounding box of the domain was made to be far larger than the discharge region, with dimensions (*r*, *z*) = [5, 3.75] mm. For the later point-point simulations, the domain was mirrored vertically, but with the needles shifted such that their tips would lie on *z* = ± 125 $\upmu$m to maintain the 250 $\upmu$m gap.

A zero-potential Dirichlet condition was applied to the bottom plane electrode (or in the case of point–point electrodes, the corresponding needle), while a time-dependent ramp voltage of the form

7 $$\begin{aligned} U_0(t) = \frac{dU}{dt}t \end{aligned}$$

was applied to the needle electrode, where the symbol *dU*/*dt* is the rate of voltage rise with units of volts per second. Neumann-zero conditions were applied to the axis of symmetry and to the outer edges, while wall conditions following Hagelaar et al.^32^ were prescribed on the electrode surfaces for all fluxes. Since the present work was focused on the working gas type and on the effects of *dU*/*dt*, no secondary emission (SE) nor reflection at the electrodes have been considered. Besides, secondary emission coefficients remain largely unknown for many engineering materials, and a systematic study on the effects of arbitrarily varying SE would be of lesser relevance in practice, as generally, SE coefficients are not well defined nor can they be controlled. It remains, however, of high priority to study in future. Apart from the uniformly-distributed electrons and positive ions representing background pre-ionization, no additional charged seeds were necessary as initial conditions, since the discharge would initiate from the enhanced field around the needle tip.

In all simulations, adaptive mesh refinement (AMR) was enabled to perform dynamic re-meshing, reaching a minimum element size of approximately 1 $\upmu$m. Time integration was of second-order accuracy, using a sub-picosecond step size, and standard pressure and temperature (STP) conditions (1 atm, 300 K) were maintained for both gases. The StrAFE library as developed and verified by Wong et al.^33^ was used to perform this study. Note that this study focused on the characteristics of only the primary ionization wave, therefore, simulations were terminated upon the wavefront reaching the opposite electrode and began to spread out laterally across its surface (or in the case of positive energization, across the cathode sheath).

## Results and discussion

In this section, the results obtained from performing the simulations are presented. It is remarked that while the term *streamer* is used during discussion, the limited dimension of the inter-electrode gap means that a *streamer* in the traditional sense of a propagating filamentary discharge is perhaps better referred to as an *ionization wave front*. This is because the thin, elongated channel characteristic of a classical *streamer* formed in longer gaps cannot be developed over such short distances. However, the term *streamer* is used interchangeably here for convenience. The first section focuses initially on the *dU*/*dt* = 50 kV/ns case only, describing aspects which were generally independent of the rate-of-rise. This includes an overview of the observed streamer morphology with comparisons between point-plane and point-point gaps. The next section presents analyses on the streamer characteristics—velocity, electric field, and the developed electron density, and how these were affected by the voltage rate-of-rise. The section “Cathode sheath” completes the results with some discussion of the cathode sheath and its behavior under differing voltage slopes.

### Ionization front morphology—point-plane and point-point

**Figure 2:**
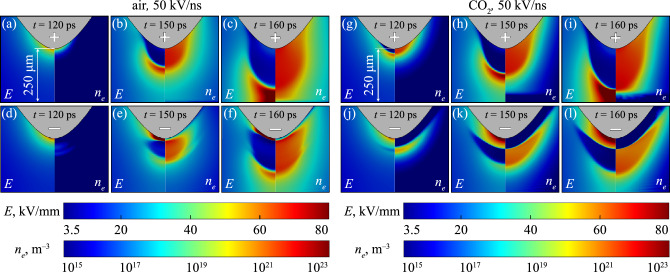
Time evolution of the ionization front in an (**a**)–(**f**) air-filled, (**g**)–(**l**) CO_2_-filled point-plane gap. Panels have been labelled with the moment in time the image was recorded, while the symbol printed on the needle electrode indicates the polarity of the applied voltage (top rows are positive, bottom rows are negative). Showing *dU*/*dt* = 50 kV/ns only.

Figure 2a–f shows the evolution of the electric field (left half of each panel) and the electron density (right half of each panel) at various times in air, near the discharge region between point-plane electrodes for the case of *dU*/*dt* = 50 kV/ns only, and for both polarities. Note that the slower rates of rise have not been shown in the main text, as the ionization fronts were morphologically identical with the exception that they were shifted in time due to the delayed initiation of the ionization wave due to the slower rising voltage. The reader is, however, directed to the additional color plots and streak images attached as Supplementary Figures S1 and S2 for a comparison of the wavefront evolution for slower rates of rise. Figure 2g–l shows the corresponding data for CO_2_. Due to the steep voltage slope, the gap becomes highly overvolted, and the primary streamer phase occurs rapidly. The time to wavefront initiation was found to be inversely proportional to *dU*/*dt* (see Fig. 5b). The time necessary to bridge the inter-electrode gap was in the range of 30–60 picoseconds (corresponding propagation velocities are discussed in a later section), which given the gap dimension, is in fair agreement with similar simulations conducted by Höft et al.^20^. For the positive case in both gases, direct inception of the ionization front at the needle tip was observed, before it grew in radius and length towards the cathode. Direct contact with the cathode does not occur due to the formation of a cathode sheath with low electron density. This is contrasted with the negative fronts, which initiates ahead of the cathode sheath now formed over the needle electrode due to initial outward electron drift. Also different from the negative case is the pre-inception behavior. Prior to the inception of a negative streamer, an initial—weakly ionizing—wave of electrons was observed to move away from the needle tip. This can be seen in Fig. 2 panel (d) at around 120 ps. As time advanced, the initial wave is consumed by a secondary wave which develops behind the first, which subsequently becomes the dominant ionization front (or streamer head) in the gap.

**Figure 3:**
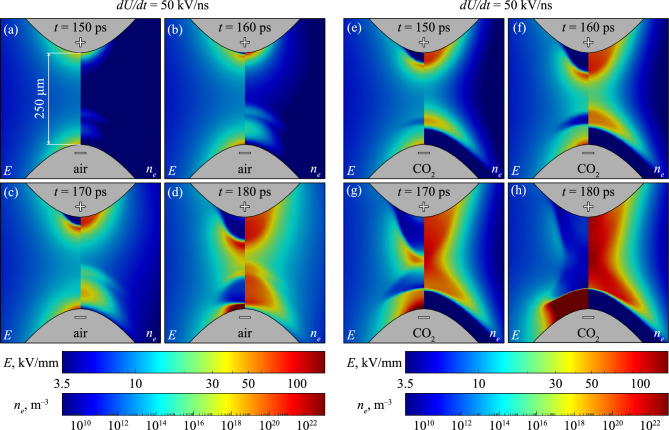
Time evolution of the ionization front in (**a**)–(**d**) air-filled, (**e**)–(**h**) CO_2_-filled point-point gap. Panels have been labelled with the moment in time the image was recorded. Showing *dU*/*dt* = 50 kV/ns, the distinction between anode and cathode is indicated by the ‘+’ and ‘–’ symbols printed on the needle electrodes.

Figure 3 shows results under the same conditions as Fig. 2 but in a point-point electrode geometry. The aforementioned phenomenon of the initial electron wave is clear in Fig. 3 panels (a) and (b), which can be seen moving away from the negative point electrode. In point-point geometries, positive and negative fronts incept almost simultaneously from the electrodes of respective polarity, which propagate and eventually collide. As was similarly observed by Höft et al.^20^, the negative front was delayed relative to the positive, likely due to the differences in the necessary field strength required for inception, which is typically higher for negative streamers^20^. This is also consistent with the results of Fig. 2, where positive fronts would incept before their negative counterparts.

In both point-plane and point-point simulations, there additionally existed a clear difference in the thickness of the cathode sheath between air and CO_2_, the dynamics of which are discussed in further detail within a later section titled “Cathode sheath”.

**Figure 4:**
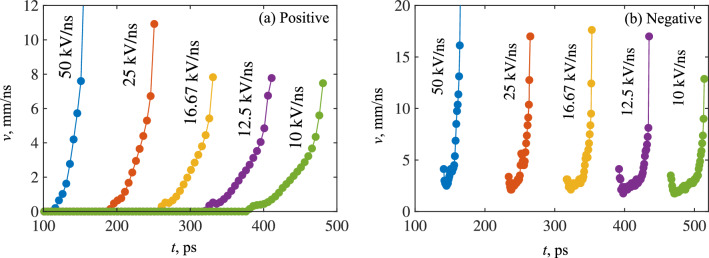
Instantaneous velocity over time, of the (**a**) positive, (**b**) negative ionization fronts in air-filled point-plane gaps for the simulated rates of voltage rise. Negative fronts also have zero velocity before the first data-point, but markers have been removed for visibility.

**Figure 5:**
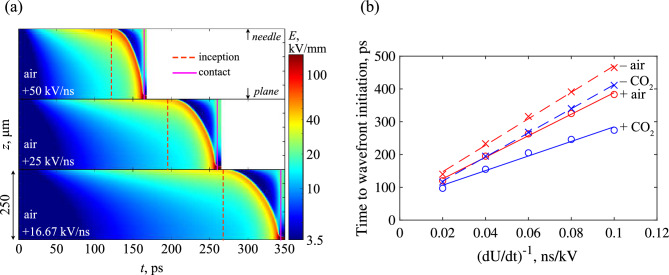
(**a**) Streak images of the electric field magnitude along the axis of symmetry for air-filled point-plane gaps, at different rates of rise. Red dotted line indicates the time of inception, solid magenta lines indicate the time of contact. (**b**) Observed linear scaling of the inception time for all combinations of gas and polarity with $(dU/dt)^{-1}$.

### Front velocity, electric field, and electron density dependency on rate of voltage rise

**Figure 6:**
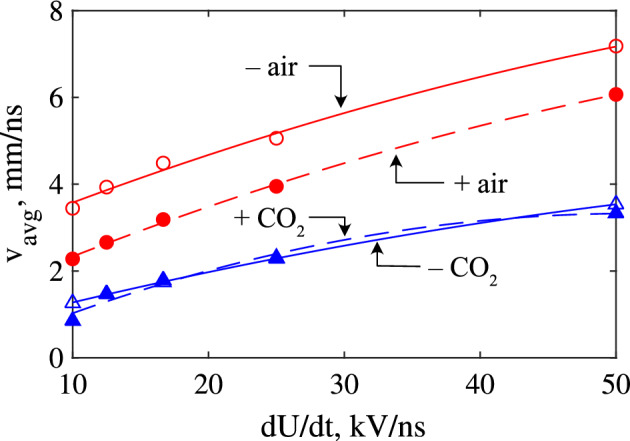
Average front velocities for air and CO_2_ under point-plane gaps, and for both polarities. Markers are simulated data, lines are best-fit curves.

The instantaneous velocities for all streamers were computed by tracking the *z* position of the point of maximum field strength along the axis of symmetry. Figure 4 shows the results in air, for both positive and negative cases, and over all simulated rates-of-rise. It should be noted that negative fronts appear to experience an abrupt change in velocity at the first plotted data point, as the negative fronts do not develop directly at the needle tip. As a result, there is an abrupt change in the position of the maximum electric field at the point of initiation, which is manifested as a sudden increase in the front velocity. In addition, negative fronts were also observed to initially decrease in velocity, corresponding to the phase when the ionization wave begins to initiate. During this phase, the plasma channel begins to develop ahead of the needle, but the front has yet to begin propagation in a self-sustained manner driven by sufficiently intense ionization at its head. Coupled with the widening of the channel due to outward electron diffusion (and consequent lowering of the electric field at its head), it is believed that these competing mechanisms may contribute to the initial decrease in propagation velocity up to the point that ionization becomes sufficient to drive the front forward. The instantaneous velocities of ionization fronts in CO_2_ evolved similarly and followed identical trends with increasing *dU*/*dt* and are therefore not shown. Overall, the velocity of all fronts grew rapidly after inception, but negative fronts appeared to experience significantly higher acceleration than their positive counterparts, and attain a higher maximum velocity during their propagation. With a slowing rate-of-rise, the effect on the maximum attained velocity is inconclusive. However, there does appear to be a reduction in the acceleration during the propagation phase—indicated by the decreasing slope of velocity with decreasing *dU*/*dt*. The computed velocities are in fair agreement with other, similar, work^20^. The average velocities were also calculated, following:

8 $$\begin{aligned} v_{avg}=\frac{d}{t_c - t_i}, \end{aligned}$$

where *d* is the distance traversed by the front (gap distance minus the cathode sheath thickness), $t_c$ is the time of contact (determined when the front ceases to have a *z* velocity, indicating that it had started to spread out over the cathode sheath in a positive case, or upon contact with the anode in the negative case), and $t_i$ is the time of inception (defined as the moment the front begins propagation, gaining a non-zero *z* velocity). The streak image shown in Fig. 5a for air under three different rates-of-rise illustrates the moments when these times were recorded, and the inverse proportionality of the inception time to *dU*/*dt* is shown for all gases and polarities in Fig. 5b. The velocities according to (8) are plotted as a function of *dU*/*dt* for both gases in Fig. 6. On average, the ionization fronts in air propagate faster than those in CO_2_. There exists a clear difference between positive and negative fronts in air, where negative fronts were, on average, consistently faster than positive fronts under the same conditions. This did not seem to be the case for CO_2_, which appeared to exhibit no significant differences in average velocity between positive and negative cases. This may be due to the far thicker cathode sheath developed during the simulated CO_2_ discharge (e.g., see Fig. 2) and its scaling with *dU*/*dt*. Despite the higher acceleration of the negative streamer—leading to a shorter time-of-flight—the effective distance traversed by the front is also reduced, such that the overall average velocity is the same as the positive case. In air, no such phenomenon existed, since, unlike CO_2_, the cathode sheath thickness was small (and did not scale with *dU*/*dt*) compared to the gap distance for all *dU*/*dt*. The cathode sheath is discussed in more detail within the section titled “Cathode sheath”. With faster rising voltage, the average velocities of all fronts increased irrespective of polarity, due to the increased background electric field developed from the greater degree of overvoltage. However, those in air appeared to exhibit a slightly greater rate of increase to average velocity relative to CO_2_. The cause of this difference may be due to differences in the electron mobility between air and CO_2_, though this requires further study.

**Figure 7:**
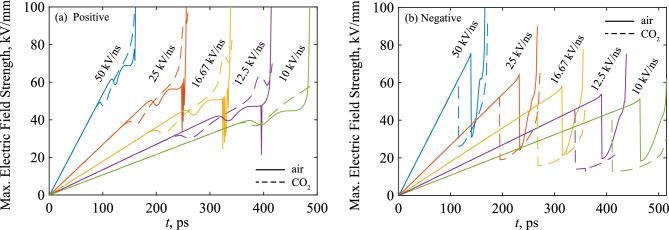
Maximum electric field over time for (**a**) positive, (**b**) negative energization, for the simulated rates-of-rise. Solid lines are for air, dashed lines are for CO_2_.

**Figure 8:**
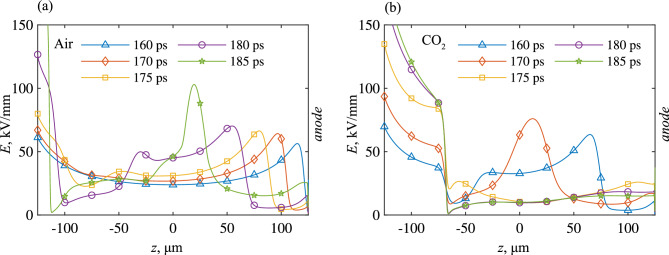
Electric field strength down the axis of symmetry for (**a**) air-filled, (**b**) CO_2_-filled point-point gaps at various timesteps. 50 kV/ns shown only.

Figure 7 shows the maximum electric field magnitude (at the streamer head) on the axis of symmetry over time between point-plane electrodes for both polarities. Initially, the electric field rises linearly, following the linearly-rising voltage applied to the electrode. In the positive case, the net field magnitude drops slightly at the moment of streamer inception as a critical charge density develops and screens the background field, before the forward propagation of the streamer is indicated by a rapid increase of the field magnitude at the streamer head. In the negative case, the maximum electric field value in the domain always remains at the needle tip due to the formation of the cathode sheath. Therefore, upon inception of the negative wavefront (ahead of the cathode sheath), the maximum electric field is instead taken as the maximum field ahead of the developed wavefront. This explains the much more significant drop in electric field magnitude in Fig. 7b. As the distance between the streamer head and the boundary decreases (on close approach to the plane electrode) the field is enhanced significantly. During this stage, some numerical oscillations were present for positive streamers (Fig. 7a), and in air only, which likely exist due to the challenge associated with resolving the thinner cathode sheath (see section named “Cathode sheath”) and steeper density gradients found in air. In point-point configurations, similar behavior was observed at both needles, i.e., the trends of Fig. 7a were observed at the anode, while those in Fig. 7b existed at the cathode. Once the two streamers initiate, the electric field is enhanced in the space between their heads, until it ultimately collapses upon the collision and combination of the two ionization fronts, as shown in Fig. 8 for air and CO_2_ under a 50 kV/ns signal. The point at which the two fronts merge is not centered in the gap (i.e., *z* = 0) due to the differing inception times and propagation velocities of the positive and negative fronts, which is in agreement with conclusions by Höft et al.^20^.

**Figure 9:**
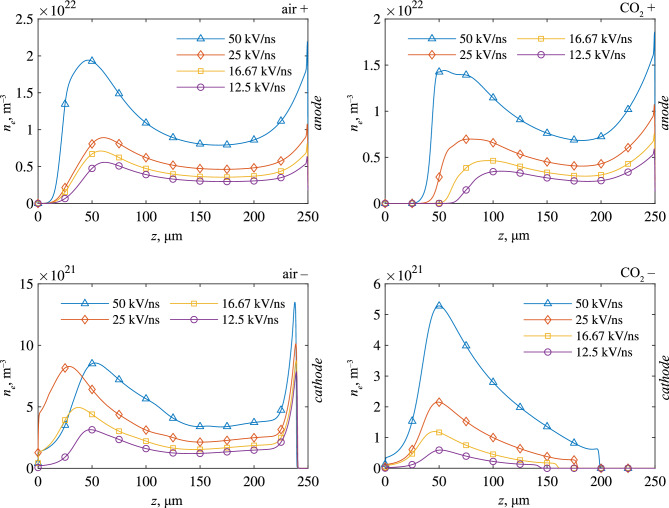
Density along the axis of symmetry at the time of contact for point-plane gaps filled with air and CO_2_. Sub-figures are labelled with the gas type (‘air’ or ‘CO_2_’), and the polarity (‘+’ or ‘–’). Plotted for *dU*/*dt* of 12.5, 16.67, 25, and 50 kV/ns.

The voltage rate-of-rise was also found to have a strong effect on the electron density developed in the resulting plasma channel. Figure 9 shows the electron density profile along the axis of symmetry at the time of contact (for point-plane) and for *dU*/*dt* = 50, 25, 16.67, and 12.5 kV/ns. In general, a positive, nonlinear correlation between the developed electron density and the voltage rate-of-rise was observed. This is once again believed to be simply due to the increased degree of overvoltage achieved at higher *dU*/*dt*, which led to the intensification of ionization and space charge production. There may also be a type of cyclic self-fulfilling behaviour here, wherein higher electric field produces increased charge densities in the channel, which in turn causes an increase in the channel conductivity, which further enhances the electric field at the front, thus further intensifying ionization. It also appears that in general, positive discharges generate far higher electron densities compared to the negative discharges. This may be linked to the maximum electric field at the streamer head, which was found to be higher for positive fronts than for negative, and has previously been linked to the more diffuse nature of negative streamers in the past^34^.

### Cathode sheath

Observed in both the point-plane and point-point studies was the development of a sheath region across the cathode. The cathode sheath is characterized by a low electron density and an intense electric field which supports much of the voltage drop across the gap, and may be formed when a discharge approaches a solid barrier, such as an electrode or solid dielectric surface^35,36^. Little is known regarding the cathode sheath, and studies have generally focused on the characteristics of discharges far from any physical boundary.

**Figure 10:**
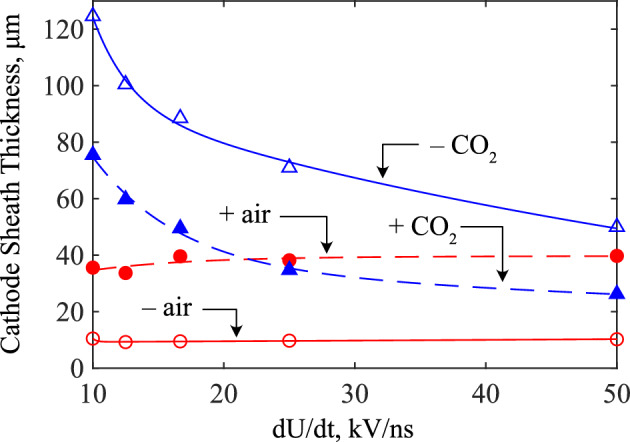
Cathode sheath thickness as a function of the voltage rate-of-rise, for fronts in air- and CO_2_-filled point-plane gaps. Markers are simulated data, lines are best-fit curves.

**Figure 11:**
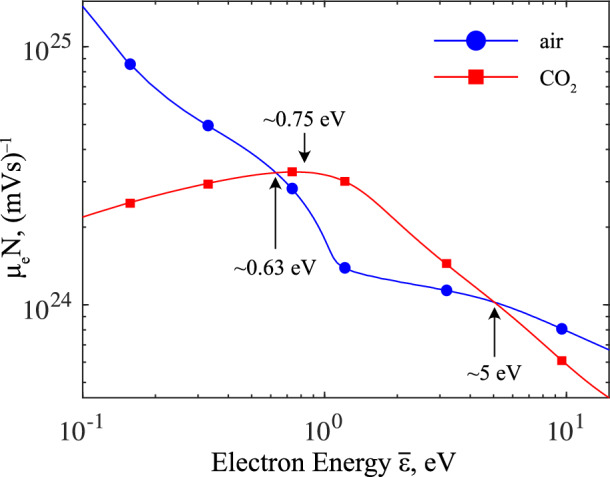
Comparison between the electronic mobility scaled by the neutral gas density as a function of the electron energy for air and CO_2_ (as computed via BOLSIG+^23^). Note the critical region where 0.63 $\le {\bar{\varepsilon }} \le$ 5 eV.

From the simulations conducted in this work, a variation in the thickness of the cathode sheath with the voltage rate-of-rise has been observed, as shown in Fig. 10. Most interestingly, this has been observed only in CO_2_ and not in air. While the color plots of Figs. 2 to 3 clearly show the formation of the cathode sheath, the regions of low electron density in Fig. 9 demonstrates more clearly the increasing thickness of the sheath with slowing rate of voltage rise in CO_2_. This can be compared to those in air, which showed very little change in the cathode sheath thickness with different *dU*/*dt*. To compare across polarities, gases, and rates-of-rise, Fig. 10 plots the cathode sheath thickness as a function of *dU*/*dt*. The physical mechanism behind this observation is believed to result from the presence of photoionization and the variation of the electron mobility with electron energy, and their impact on electron generation and drift under a time-increasing electric field during the pre-discharge phase. Figure 11 shows the electron mobility relationships for air and CO_2_, plotted against the electron energy. Of particular interest is the region where 0.63 $\lesssim \bar{\varepsilon } \lesssim$ 5 eV, where the electron mobility of CO_2_ increases to a local maximum, and becomes significantly higher than that of air. It is important to note that the simulations indicate that electrons may enter this energy range while the electric field remained below the critical field for both CO_2_ and air, at $\sim$2.2 kV/mm and $\sim$ 2.8 kV/mm, respectively. With a voltage (and hence background field) which has a finite time to rise, there must exist a duration when the local mean electron energy passes through the above energy range. During this time, electron mobility is maximized in CO_2_, allowing electrons to drift away from the cathode faster than in air, leading to the local reduction of the electron density around the cathode which eventually becomes the cathode sheath, all which occurs before intensive ionization takes place. It follows, therefore, that a slower rate of rise prolongs the time for which the field is within this critical range, allowing electrons to drift farther away from the cathode, and forming a larger sheath with slower rates of rise. The presence of significant photoionization in air compared to CO_2_ may also generate significant numbers of electrons ahead of the wavefront and injected into the sheath region, effectively decreasing the sheath thickness. For both needle-plane and needle-needle simulations, and at the slowest rate of rise simulated in this work (10 kV/ns), the cathode sheath in CO_2_ occupied almost half of the total inter-electrode gap distance. If this is indeed the case, one should expect that for sufficiently slow rising voltages (possibly kV/$\upmu$s, kV/ms), a similar relation would be found in air since the electron mobility in air is maximized at low electron energies. This would require significantly longer simulation times, as such, is considered an aspect for future work. It is believed that it was not observed in the present work due to the rapid rates of voltage rise used in this study, and with the monotonically decreasing electron mobility with increasing field in air, any differences in the electron traversal distance under different values of *dU*/*dt* during the pre-discharge phase would be indiscernible. It is further remarked that one should not ignore the possibility that other processes (attachment, recombination within the channel, electron emission from the electrodes, etc.) may also contribute to the cathode sheath behavior. These are aspects that would be of high interest to explore in further modelling and experimental work, but fall outside of the scope of the present study.

**Figure 12:**
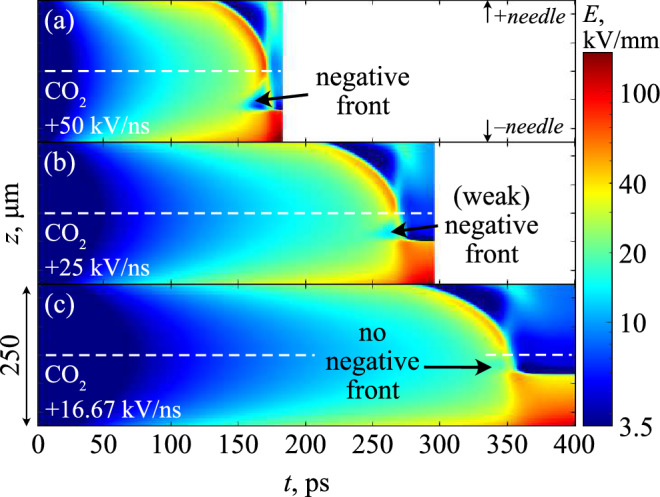
Streak image of the electric field magnitude along the axis of symmetry for a CO_2_-filled point-point gap, under different rates of rise. Dashed white line shows the midpoint between the two needle electrodes. Negative front did not form for *dU*/*dt* less than 25 kV/ns, as indicated.

It is believed that the dynamics of the cathode sheath observed here may have consequences for the discharge evolution in short, sub-mm gaps. For example, Fig. 12 shows streak images of the electric field for point-point discharges at various rates-of-rise in CO_2_. As described in the section “Ionization front morphology—point-plane and point-point”, near-simultaneous positive and negative streamers propagate towards each other and merge within the gap. This was true for all *dU*/*dt* except when *dU*/*dt* was less than 25 kV/ns in CO_2_. Under these conditions, it was found that a negative streamer simply did not appear in CO_2_ (Fig. 12). This appears to be due to the aforementioned relationship between low *dU*/*dt* and the cathode sheath, which under these conditions occupied a significant portion of the inter-electrode gap, suppressing the formation of a negative front due to the limited space. The time evolution therefore resembled more of the inception of a solitary positive streamer, which collided and spread out over a now large cathode sheath acting like an extended virtual cathode. These results may help to possibly explain some observations in short, 100 $\upmu$m needle-plane breakdown studies under impulsive regimes, conducted by Liu et al.^11^. The authors consistently found a significantly shorter time-to-breakdown at higher breakdown voltage in CO_2_ compared to air and N_2_ for nanosecond impulses, contrasting what the critical field value would suggest under classical breakdown theories. It is believed that the combination of the sub-mm gap and the enhanced electron mobility of CO_2_ during the rising impulse may act to significantly reduce the electron density in the gap during the pre-discharge phase. This may inhibit the formation of initial avalanches and ionization fronts, thereby delaying the breakdown process and increasing the overall breakdown strength. It is thought that such phenomena would be more difficult to observe in long gaps, as the field would have to remain within the critical range of enhanced electron mobility for far longer to cause a significant reduction in the electron density ahead of the streamer. That is, the increase of the breakdown strength for CO_2_ only holds when the sheath distance is able to occupy a significant proportion of the inter-electrode gap. Of course, the simulations here omit other possible electronic sources, such as charge injection at the electrodes or any secondary emission sources, and forgoes the consideration of statistical processes before—and other processes beyond—the primary ionization front. Comparison with practical breakdown data is therefore done with extreme caution, and would necessitate significant experimental and theoretical work in the future to confirm the existence of the discussed mechanisms.

## Conclusions

In this work, the computational study of fast transient ionization fronts initiated in sub-millimeter point-plane and point-point gaps has been performed. Analysis has been conducted on the effects of fast-rising ramp (over)voltages on the primary discharge characteristics in synthetic air and in pure CO_2_. It has been observed that ionization fronts initiated in air develops stronger electric fields at their heads than in CO_2_ for negative voltages, but the opposite behavior is found for positive voltages. Ionization fronts developed in CO_2_ also appear to be larger in radius and incept earlier than in air under the same conditions. The acceleration of the ionization fronts is affected by the voltage rate-of-rise, where slower rising voltages led to slower-accelerating fronts, while average propagation velocities increased with increased voltage rate-of-rise, through this was more significant in air than in CO_2_.

The developed electron density was higher for positive fronts than for negative fronts in the same gas, under the same conditions of voltage stress, and an increased rate of voltage rise increased the electron density developed inside the resulting plasma channel nonlinearly. By decreasing the rate of voltage rise, a thicker cathode sheath was developed in CO_2_, but little change to the cathode sheath in air was observed. This is believed to be due to the enhanced electronic mobility of CO_2_ for a specific range of electron energy during the rising edge, and the presence of photoionization in air. It has been hypothesized that air would exhibit similar behaviour for voltages rising slower than those used in this study. The relationship between the rate-of-rise and the cathode sheath thickness may have consequences for the operation of systems utilizing short gaps. In a point-point simulation, this phenomenon is believed to have suppressed the formation of a negative front (and early halting of the positive front). It is believed that the ratio between the sheath thickness and the total inter-electrode gap distance is an important parameter in characterizing this process, as is the voltage rate-of-rise.

In future work, further investigation of the cathode sheath development, and its relation to time-varying voltages, would be of great interest. In particular, further simulations with different gases, and under slower rates-of-rise would be necessary to fully understand the mechanism behind the observed sheath effects. Experimental work on the breakdown of ‘pre-stressed’ sub-mm gaps should also be conducted, to further understand whether the effects observed here have any tangible impact on the overall breakdown process. This could potentially be conducted using superimposed DC and impulsive voltages. Studies beyond the initial primary discharge phase would also be of great importance, especially to assess the impact of the primary ionization front on the further evolution of the discharge through to the spark stage. Developments in this direction would contribute to a deeper understanding of gas discharge processes, and be highly beneficial to the design of devices dealing with high electric field stresses, including high voltage and pulsed power equipment.

### Supplementary Information

Supplementary Information.

## Data Availability

All data generated or analyzed during this study are included in this published article (and its Supplementary Information files).
