# Supplementary material for: A computational study on the effects of fast-rising voltage on ionization fronts initiated in sub-mm air and CO2 gaps
Source: Sci Rep. 2024 Jan 12;14:1185. doi: 10.1038/s41598-024-51727-y (PMC10786894; doi:10.1038/s41598-024-51727-y)
Supplement: Supplementary file 1 — Supplementary Information. [file 41598_2024_51727_MOESM1_ESM.pdf]

A Computational Study on the Effects of Fast-rising Voltage on Ionization Fronts Initiated in Sub-mm Air and CO_2 Gaps

T. Wong, I. Timoshkin, S. MacGregor, M. Wilson, and M. Given,

Supplementary Materials

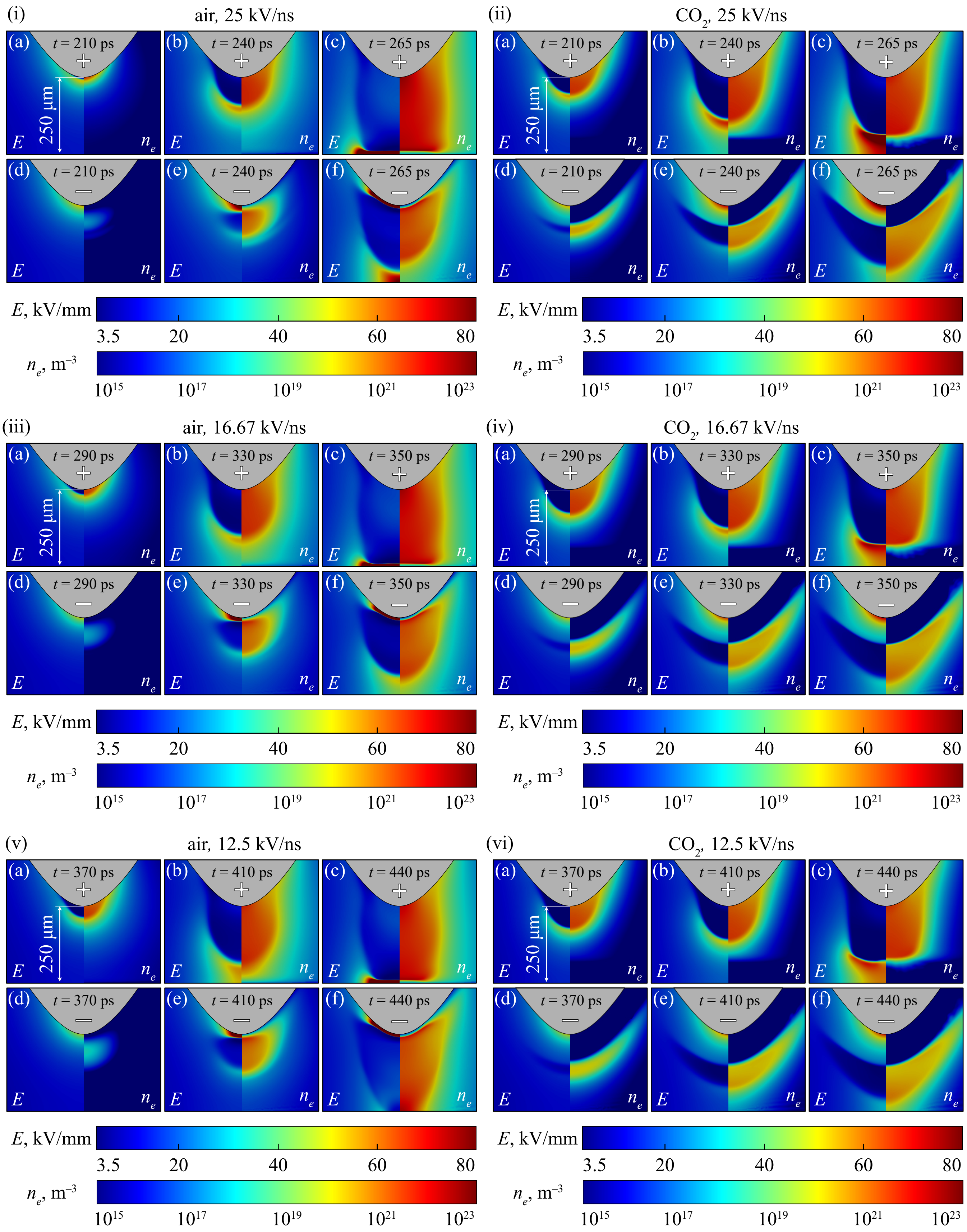

Figure S1: Colour plots of electric field and electron density for air (left col.) and CO_2 (right col.) for needle-plane cases, for slower rates of rise. Shows the similarity in the wavefront development, just delayed in time.

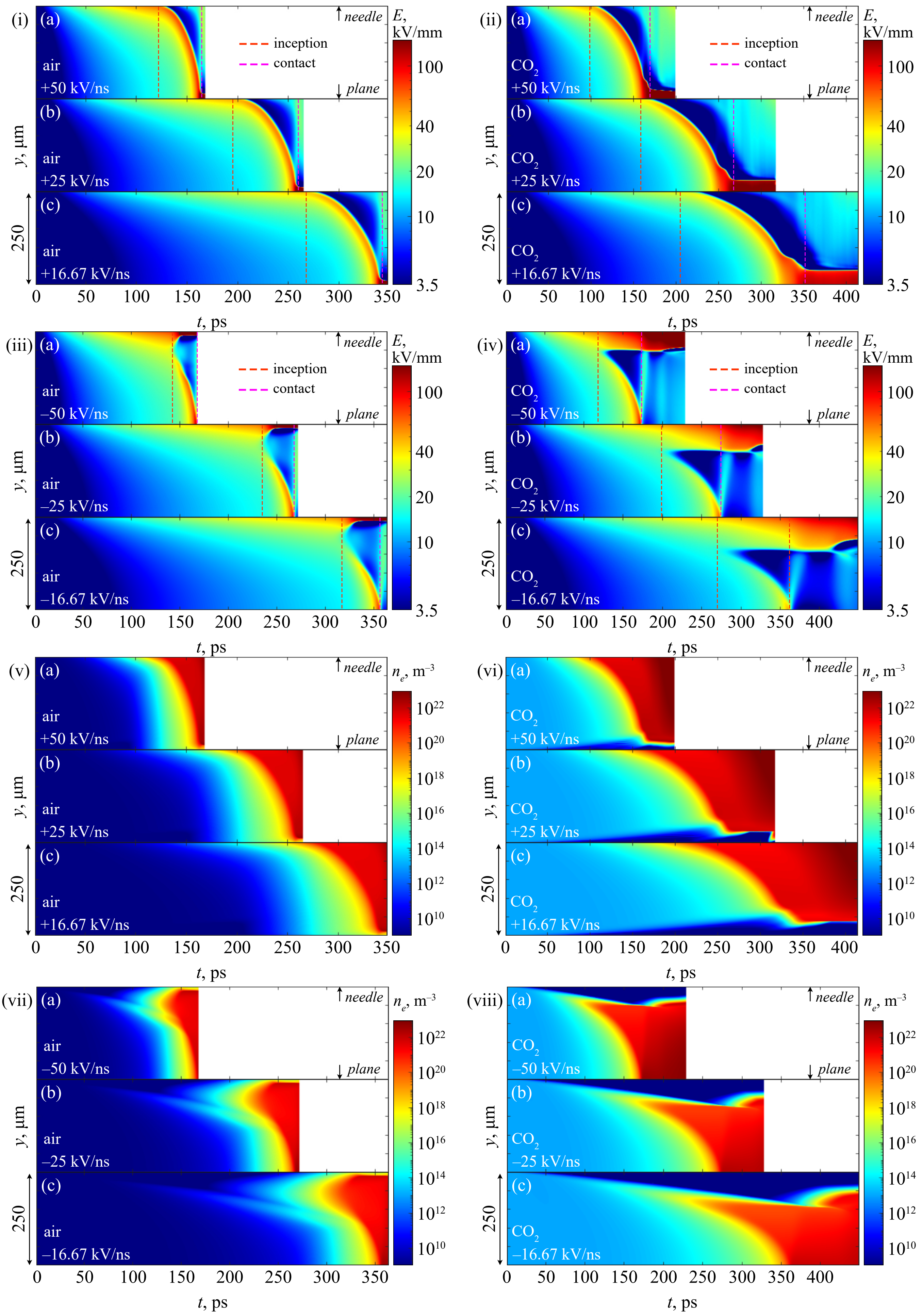

Figure S2: Streak images of electric field [(i)-(iv)] and electron density [(v)-(viii)] for air (left col.) and CO_2 (right col.) for needle-plane cases, for different rates of rise. Shows the similarity in the wavefront development, just delayed in time.
